# Difficulties associated with outpatient management of drug abusers by general practitioners. A cross-sectional survey of general practitioners with and without methadone patients in Switzerland

**DOI:** 10.1186/1471-2296-6-51

**Published:** 2005-12-19

**Authors:** Anne Pelet, Jacques Besson, Alain Pécoud, Bernard Favrat

**Affiliations:** 1 Policlinique Médicale Universitaire, Lausanne, Switzerland; 2 Centre Saint-Martin, Département de Psychiatrie Universitaire de l'Adulte, Rue Saint-Martin 7, 1003 Lausanne, Switzerland

## Abstract

**Background:**

In Switzerland, general practitioners (GPs) manage most of the patients receiving methadone maintenance treatment (MMT).

**Methods:**

Using a cross-sectional postal survey of GPs who treat MMT patients and GPs who do not, we studied the difficulties encountered in the out-patient management of drug-addicted patients. We sent a questionnaire to every GP with MMT patients (556) in the French-speaking part of Switzerland (1,757,000 inhabitants). We sent another shorter questionnaire to primary care physicians without MMT patients living in the Swiss Canton of Vaud.

**Results:**

The response rate was 63.3%. The highest methadone dose given by GPs to MMT patients averaged 120.4 mg/day. When asked about help they would like to be given, GPs with MMT patients primarily mentioned the importance of receiving adequate fees for the care they provide. Secondly, they mentioned the importance of better training, better knowledge of psychiatric pathologies, and discussion groups on practical cases. GPs without MMT patients refuse to treat these patients mostly for emotional and relational reasons.

**Conclusion:**

GPs encounter financial, relational and emotional difficulties with MMT patients. They desire better fees for services and better training.

## Background

Methadone maintenance treatment (MMT) is extensively used for opiate addiction. Providing care in an office-based practice is feasible [1,2] and produces outcomes comparable to those from specialist treatment [1,3-7]. Furthermore, it reduces the stigma associated with the diagnosis and treatment of substance abuse and increases the amount of attention paid to medical and psychiatric conditions [3,4]. Easy geographical access to treatment encourages employment rehabilitation and retention in treatment [4]. However, GPs encounter specific difficulties with this population: burnout, lack of training, a negative attitude and a lack of motivation have been widely reported [3,8,9]. These difficulties prevent some GPs from accepting MMT patients.

Furthermore, each country manages MMT in a different way: the UK encourages every general practitioner to prescribe methadone through national policies and guidelines; France usually reserves MMT for specialized centers and promotes the use of buprenorphine [10]; and the United States only recently began to allow GPs to prescribe MMT.

In Switzerland, most patients on MMT are treated by GPs, currently using the oral liquid form of methadone. Buprenorphine use is very rare and codeine is not encouraged. Only some specialized centers treat opiate abusers with injectable heroin. GPs have to register for every opiate substitution with methadone to afford the double prescription.

The Swiss government encourages substitution treatment for drug-addicted individuals in the context of a harm-reduction policy, but does not push GPs to accept these patients and has never distributed national guidelines broadly as has the UK. Generally, however, Swiss GPs are used to providing pharmacotherapies and other treatments to drug users in our country. There is no shared care as in England, but Swiss GPs use a pragmatic approach, including meeting with social workers and pharmacists in charge of MMT patients. Groups of GPs involved with the drug-addicted have been created and receive support from the Federal Office of Public Health for continuous formation and clinical discussion.

Specialized centers with psychiatrists, social workers, psychologists and medical doctors offer multidisciplinary management for unstable patients. However, in Switzerland as elsewhere, there are not enough specialized centers for all drug addicts requiring treatment, and access is limited by geographical barriers and the restricted number of treatment places that can be offered [11]. Furthermore, some geographical regions do not have specialized centers.

Office-based treatment provides clear advantages for drug-abusing patients. However, primary care practitioners encounter specific difficulties with this patient population. Burnout, lack of training, a negative attitude and a lack of motivation have been reported widely among the GP population [3,8,9]. These difficulties discourage and prevent primary care physicians from accepting drug abusers for substitution treatment.

The aims of this study were to (1) to describe the specific difficulties with the MMT population encountered by primary care physicians and to identify why primary care physicians are reluctant to manage drug-abusing patients in Switzerland; and (2) to identify primary care physicians' needs in terms of future management of drug-abusing patients in the French-speaking part of Switzerland and to suggest solutions to help them.

## Methods

We mailed a multiple-choice questionnaire designed to evaluate various aspects of the difficulties encountered in treating MMT patients: pharmacological issues (highest methadone dose), legal requirements, financial issues (how GPs get paid), emotional and psychiatric aspects (including psychiatric medication and referral to a psychiatrist), relationships, multidisciplinary interactions (e.g. with social workers or with pharmacists), motivation of primary care physicians, and specific management in the office setting. We collected the material to develop this questionnaire during semi-formal interviews with the staff at Saint-Martin (a specialized center for managing drug abusers). MedRoTox practitioners (a group of general practitioners concerned with the problem of dependency and supported by the Swiss Federal Office of Public Health) reviewed the questionnaire. During the calendar year 2000, we mailed it for anonymous completion to every primary care practitioner with MMT patients in the French-speaking part of Switzerland (556 physicians). This figure includes every GP prescribing methadone in this part of the country, which has a population of 1,757,000. We sent the questionnaire again three months later to increase the response rate and received answers over the following three months.

We sent another questionnaire to GPs without MMT patients to evaluate more specifically the factors that kept them from accepting these patients. We used the mailing list kept by the outpatient clinic at Lausanne University Hospital. These 365 GPs represent most of the primary care practitioners in the Swiss canton of Vaud. This questionnaire, also for anonymous completion, was also sent twice at an interval of three months.

Both questionnaires are available from the corresponding author.

We used descriptive statistics and the chi-square test for comparison. We performed all statistical analyses by SPSS software, version 11.

## Results

Of the 556 targeted GPs with MMT patients (PT: practitioners with MMT patients), 63.3% (352) responded. We received replies from 231 (63%) of the targeted 365 primary care physicians without MMT patients (PWT: practitioners without MMT patients). Table 1 shows the profiles of both groups of GPs.

**Table 1:** Profile of office-based physicians with and without methadone substitution treatment

	PT (n = 352)	PWT (n = 231)	X^2^, p value
Male	84.9%	82.7%	X^2 ^= 0.4; p = 0.563
Practice in a town with >10,000 inhabitants	60%	66.2%	
Practice in a town with <10,000 inhabitants	40%	33.8%	X^2 ^= 2.3; p = 0.136
Works alone	57.9%	65.7%	
Works in a group practice (with other doctors)	42.1%	34.3%	X^2 ^= 3.5; p = 0.068
Median no. of years in practice* (mean SD)	15 (14.8 ± 7.4)	17 (17 ± 8.6)	Z = -3.2; p = 0.001

PT = General practitioners with patients receiving methadone substitution treatment

PWT = General practitioners without patients receiving methadone substitution treatment

*P value was calculated using the Mann-Whitney test; other P values were calculated using Fisher's exact test

Both populations (PT and PWT) were similar in terms of gender frequencies, practice location and the percentage who work in a group practice. The only statistically significant difference was the mean number of years in medical practice (PT: 14.8 years, PWT: 17 years).

Table 2: Of the total PTs respondents, 73% would not accept more patients. The mean number (± SD) of MMT patients that a PT would like to have (5.8 ± 6.9, median 4) was slightly less than the mean number that they actually treat (mean: 6.2 ± 9.04, median 4). Responding PTs reported an average highest daily methadone dose of 120.4 mg/day (median = 100 mg/day, mode = 100 mg).

**Table 2:** General practitioners who treat patients with methadone substitution (PT, n = 352)

Would you accept new MMT patients?	Yes: 23.9 %	No: 73% (missing data: 3.1%)
	Mean (SD)	Median

Actual number of patients on methadone treatment?	6.2 (± 9.04)	4
How many patients would you *like *to have on MMT?	5.8 (± 6.9)	4
Highest daily dose of methadone you ever prescribed:	120.4 mg (± 95.9)	100 mg (mode: 100 mg)

PT = General practitioners with patients receiving methadone substitution treatment

PWT = General practitioners without patients receiving methadone substitution treatment

MMT = Methadone maintenance treatment

The percentage of PWTs who had received requests for methadone treatment was 52%. Of the responding PWTs, 42.9% had been involved in methadone treatment in the past but were no longer treating such patients. Of the PWTs, 88.7% did not treat MMT patients because they refused to accept them into their practice.

Table 3 shows the improvements reported by both PTs and PWTs as necessary for improving MMT patient management. PTs mostly emphasized better reimbursement for related items of service. They also frequently mentioned better training (post-graduate or specialized psychiatric training) and more interaction with other professionals, including groups for discussing clinical cases. PWTs gave priority to having more centers and more specialized professionals for treating drug-addicted patients.

**Table 3:** What could be done to improve the management of patients on methadone maintenance treatment (MMT)?

Question:	PT (n = 352)	PWT (n = 231)	X^2^	P
Better reimbursement for care	58.8%	Question not asked		
Better post-graduate training	50%	31.2%	56.9	< 0.001
Group discussion of clinical cases	50%	22.9%	42.8	< 0.001
Better knowledge of psychiatric pathologies	46.6%	21.2%	38.7	< 0.001
More political commitment to drug-addicted patients (more centers, more specialized professionals)	44.6%	64.1%	21.2	< 0.001
More accessible specialized professionals	42%	37.2%	1.3	0.262
Better training at medical school	41.2%	19%	6.0	0.014
Better screening for drug addiction by private practitioners	18.2%	16.5%	0.3	0.656
Stronger political repression of illegal drugs	9.7%	19.5%	11.5	0.001

PT = General practitioners with patients on methadone substitution treatment

PWT = General practitioners without patients on methadone substitution treatment

Of the PT group, 56% of physicians reported difficulties with medical care reimbursement (table 4). Also, a total of 76.1% had learned about MMT through their own practices rather than receiving formal training. Most PTs are interested in investing time in further training.

**Table 4:** Questions about reimbursement and training for general practitioners who treat patients with methadone substitution (PT: n = 352)

Question		yes
Do you have problems getting medical care reimbursed?		56.7%
How did you learn about MMT	learning through their own practice?	76.1%
	post-graduate training?	58%
	self-taught? (books or scientific articles)	49.7%
	during your internship?	15.6%
Would you like to participate in addiction-related training for doctors?		65.3%
Would you like to participate in workshops with physicians and others professionals involved in the field?		50%

PT = General practitioners with patients receiving methadone substitution treatment

MMT = Methadone maintenance treatment

Table 5 shows reasons why PWTs refuse MMT patients. PWTs rated non-compliant patients as the biggest obstacle to management (59.7%) and preferred patients to be managed by a specialized center (57.1%). The "time-consuming" nature of treatment for drug-addicted patients was another major reason (54.5%) cited for not accepting them.

**Table 5:** Why do you refuse care for methadone treatment patients (general practitioners with no patients on methadone substitution treatment) (PWTs) (n = 231)

Question	yes
Non-compliant patients	59.7%
A specialized center is better	57.1%
Time-consuming	54.5%
Lack of specialized training	52.4%
Fear of being overwhelmed by drug-addicted patients	48.9%
Feeling of powerlessness toward drug-addicted patients	41.6%
Difficulties with authorizations (too much paperwork)	40.3%
Lack of reimbursement for medical care	38.1%
Management with other professionals too difficult	38.1%
Difficulties with accompanying psychiatric disorders	36.4%
Fear of manipulation by drug-addicted patients	33.3%
Lack of knowledge about illegal substances and medications	31.2%
Past experience of burnout with drug-addicted patients	29.4%
Fear of theft	28.1%
Fear of problems with other, non-drug-addicted patients	27.7%
Fear of being threatened in the office	24.7%
Fear of being considered "the drug-addict doctor"	11.7%
Existing centers are adequate for this population	11.3%

PWT = General practitioners without patients receiving methadone substitution treatment

## Discussion

The disinclination of the PWTs to treat MMT patients (88.7%) raises the question of how to change this attitude, especially in light of the growing need for MMT (9,700 patients treated with methadone in 1991 and 15,382 in 1997 in Switzerland) [12] and the lack of specialized centers and government policies encouraging easy access to MMT. More medical training, specific training during residency and the development of faculty role models would probably contribute to improving attitudes [7]. A strikingly high percentage of GPs refuse to treat MMT patients for reasons linked to relationships or management of emotions (noncompliant patients, fears, feeling of powerlessness, or burnout in the past, as illustrated at Table 5). Again, better role models, a more positive attitude during basic medical training and greater emotional and professional support for practitioners involved with MMT patients could perhaps overcome these barriers. Of the PWTs, 11.3 % would still accept MMT patients. This information suggests an area warranting further research, and we need better tools to identify, reach, teach and encourage these physicians.

The highest average daily dose of methadone (120.4 mg/day, table 2) is not surprising in view of the recommendations in the literature: although daily doses of methadone may differ from one patient to another, some authors recommend daily doses between 60 and 100 mg/day [15-19]. A UK postal survey addressed to GPs in 2001 identified a mean methadone dose of 36.9 mg [14]; although this information (the mean) differs from the information obtained in our study (highest methadone dose prescribed), our result is still higher than expected: generally, GPs are known to prescribe low doses of methadone, contrary to international recommendations [14,16]. However, Swiss GPs with MMT patients seem to be more aware of these recommendations. We hypothesize that PTs may have better formation and could be more concerned about methadone issues.

When asked how the management of MMT patients could be improved (table 3), PTs first mentioned better reimbursement for services provided. In Switzerland, patients pay part of the costs of health care, with the mandatory health insurance system picking up the rest of the bill. In some instances, health insurance companies reimburse patients so that they can pay their physicians directly. This practice was intended to create a stronger sense of responsibility among patients for the cost of their medical treatment. However, it is often difficult for a drug-addicted patient to reimburse physicians with these funds; the drug-addicted usually have difficulties with money management and may spend the money instead on illicit drugs. If the patient does not pay, money is rapidly deducted from the social help provided to pay the monthly health insurance. But the physician may not receive the portion for which the patient is responsible. This payment system is specific to Switzerland but shows that adequate reimbursement is important.

Weinrich and Stuart have demonstrated that professional and financial help are crucial for primary care practitioners in Scotland [2]. In the UK, financial rewards for general practitioners helped them to accept and continue working with MMT patients [2,14].

In contrast, PWTs suggest increasing the number of specialized centers and developing more accessible specialized professional help as the first steps towards improving MMT patient management (table 5). These suggestions are fully in keeping with their attitudes about not accepting MMT patients. As the results showed, significantly more PTs than PWTs felt that better postgraduate training could improve the management of patients in MMT.

PTs mentioned lack of training as the second area for improvement in the management of MMT patients, suggesting better postgraduate formation, discussion groups focusing on clinical cases, better knowledge of psychiatric pathologies, and better training during residency. The need of GPs for adequate training in addiction is well known [9]. Miller et al. [13] emphasized the lack of specific training at medical schools and the absence of a positive attitude and role models among faculty and physicians. In the French-speaking part of Switzerland, two universities have medical schools. One (the University of Lausanne) offers a 12-hour teaching module including alcohol- and drug-related dependence and one day of practice in the psychiatric service; the other (the University of Geneva) has 80 hours of teaching on alcohol- and drug-related problems. However, this formal training in addiction began only a few years ago. Each region also has its own training opportunities, depending on the local network. Although local discussion groups for clinical cases already exist, the high percentage of PTs who expressed a need for more training points to a current overall lack of training.

Our study found that 76.1% of PTs have learned through their own daily practice how to manage methadone treatment (table 4). This is another powerful illustration of the lack of training that doctors receive and makes an urgent case for the development of better training opportunities for primary care practitioners who provide methadone treatment in Switzerland.

Two concerns mentioned with similar frequency by PTs were the need for more political support in the treatment of drug-addicted patients (provision of more centers, more specialized professionals) and the need for more accessible specialists (table 3). Interestingly, PTs are more interested in improving their own practices (through reimbursement of fees and training) than in developing other infrastructure for treating drug abusers. This finding illustrates the concentration of MMT patient treatment in the outpatient setting in Switzerland.

The median number of patients managed by the PTs represented in the survey is fairly low (four per practitioner, table 2) but is comparable with a recent postal survey in England (3.58 patients with opiate substitution per prescribing GP) [14]. However, when asked how many MMT patients they would like to treat, PTs responded with an even lower number. This finding underlines the limited capacity of a single physician to accept and treat MMT patients and the burden represented by these patients. In Switzerland, the primary care practitioners who accept patients for methadone treatment probably represent those doctors who are more trained and more interested in MMT patients.

Our study has some limitations. First, the PWT population was sampled from the Canton of Vaud. We intentionally chose this Canton because it includes both an urban and a rural population. Both survey populations (PT and PWT) were similar in terms of sex, location in a village or a town (>10,000 inhabitants) and the type of practice (single or shared). The only statistically significant difference was the mean number of years of practice in the two populations. Age and training in treating drug misuse have been shown to affect attitude (9); thus, younger practitioners may be more likely to consider MMT patients as medical patients than as stigmatized individuals. Furthermore, older practitioners were not as accustomed to delivering methadone.

The response rate to the survey (63.3% of PT and 63% of PWT) is another limitation. Although it is a high rate for a nine-page questionnaire, the survey still represents the opinions of only some practitioners. Compared with the literature, it was better than expected from a busy general practice [20]. Even if the return rate was similar for PTs and PWTs, there could be a bias of greater interest in the subject or of greater personal involvement.

A final limitation is the fact that the questionnaire was not formally validated by inter-rater techniques.

## Conclusion

Each country has specific needs and characteristics for drug management, depending on government policies and the existing health care network. Switzerland has a policy of decentralization and harm reduction based on a low-threshold approach and a broad access to MMT through GPs, a policy embroiled in major political and emotional controversies. "Shared care" only exists in specialized centers in the form of multidisciplinary work with drug nurses, psychiatrists, social workers and GPs, but is reserved for more disruptive and unstable patients. In Switerzland, MMT prescribed outside specialized centers involves only a highly selected group of GPs trained in the addiction field. National policies, however, encourage GPs to work in multidisciplinary teams and to meet regularly with social workers and other healthcare providers.

If the intention is specifically to achieve a low-threshold approach through generalizing MMT prescription, we need to listen to suggestions made by the principal players, i.e. the general practitioners. PTs want reimbursement for their services and better training. The growing needs of drug-addicted patients, the spread of HIV and the greater emphasis on harm-reduction policies are surely powerful reasons for answering this plea and providing support to practitioners who accept MMT patients.

## List of abbreviations used

GP: General Practitioners

MMT: Methadone maintenance treatment

PT: Practitioners with patients on methadone maintenance treatment

PWT: Practitioners without patients on methadone maintenance treatment

## Competing interests

The author(s) declare that they have no competing interests.

## Authors' contributions

A. Pelet designed the questionnaires, performed the statistical analysis and drafted the manuscript. A. Pécoud provided input on interpretations discussed in the manuscript and aided in manuscript preparation. JB helped with study design and manuscript preparation. BF participated in the design and coordination of the study, helped to perform the statistical analysis and helped to draft the manuscript.

## Pre-publication history

The pre-publication history for this paper can be accessed here:


